# Some Advice to Give, and Some Not to Give, the Tuberculous

**Published:** 1904-09

**Authors:** Arch Dixon

**Affiliations:** El Paso, Texas


### SOME ADVICE TO GIVE, AND SOME NOT TO GIVE, THE TUBERCULOUS.

BY ARCH DIXON, JR., M.D., EL PASO, TEXAS.

For a number of years the subject of pulmonary tuberculosis has received more attention than any other medical topic. Medical journals have been full of articles on its cause, prevention and cure; the medical societies have discussed it at length; societies have been formed to stamp it out; and even the lay press has disseminated valuable knowledge on the subject. But, in spite of this—if testimony of patients is to be believed, and there is so much of it, and of such uniformity that it can not be doubted—there is, in the profession to-day, a surprisingly large number of men who do not know the first principles of the modern treatment of the disease. Patients are still sent West without the slightest regard being paid to their condition, and the elevation they are being sent to, with their pockets stuffed with prescriptions for stomach-destroying cough mixtures, and with the same old instructions to rough it, take as much exercise as possible, get on a ranch and hunt and ride, stay away from doctors, drink whisky, etc. Not only that, but hopelessly ill third-stage cases are still being sent West to die, many of them with hardly sufficient means to procure the bare necessities of life, much less the luxuries of which they stand so much in need. And it is against these practices I want to protest.

No tuberculous subject should be advised to leave home in search of climate unless he, or she, is financially able to be idle at least four months. Cases handicapped by having to earn a livelihood from the start have an extremely uphill fight to make, and rarely do well. The crucial time for these unfortunates is during the first few months after the diagnosis is made, and during that time they should devote their entire time to the effort to regain their health.

It is a mere truism to say that the chance of a tuberculous case regaining his health is in direct proportion to the earliness of the diagnosis and the promptness of the institution of proper treatment. It should, therefore, be the rule to advise these cases to change climate at the earliest possible moment, when the chances of recovery are so much greater than when the disease has obtained a firm foothold. Tuberculosis is called by many names, but no other disease is ever called tuberculosis; at least not in this country, and in making a diagnosis, the safe thing is to look on suspicious cases as tubercular until proven otherwise.

It is a mistake to tell a tuberculous person that his lungs are weak, or that if he does not change climate he will probably develop the disease. It is much better to frankly tell him that he has the disease, for otherwise it will be impossible to induce him to carry out the necessary treatment. Telling a patient he has tuberculosis is no longer condemning him to death, as the disease is remarkably curable, if discovered early, and proper treatment instituted, and saving a patient's feelings at the expense of losing the best opportunity of curing him is radically wrong.

Tuberculous cases who are advised to come West should not be told that they will have to remain a few months or a year, and they can then return home in safety. It is much better to tell them in the beginning that in all probability they will have to go for good and all, as there is no safety for a tuberculous case in the climate in which the disease was developed. And once they know they must live West, they are much better contented than when they are living in constant expectation of returning home. A few cases return East after having recovered in the West, and the recovery remains permanent, but the number is extremely small. Any phthisio-therapeutist in the Southwest will bear me out in the statement that tuberculous cases by the score recover, at least, clinically, in this section only to return East and redevelop the disease—a proof positive of the great advantages of a suitable climate in the treatment of this most fatal but one, of all diseases.

Tuberculous cases should not be advised to live in a tent unless they are also instructed to always leave one end of the tent open for ventilation. Tent life, as practiced by the average health-seeker, is a boomerang. Little or no air goes through good canvas, yet many tuberculous tent-dwellers keep their tents tightly closed, spending a good part of the day, as well as the night, in them, fondly deluded with the idea that they are breathing nothing but fresh air. Some of these poor unfortunates, not content with the amount of harm they do themselves, living in a tight tent, must needs hasten their destruction by the use of one of those death-dealing devices—an oil stove. Such a combination—an oil stove and a tight tent—will kill a tuberculous subject with greater promptness and dispatch than any other I know of. A good-sized room, with at least two windows, and the windows on hinges so they can be opened like a door, is much preferable to a tent, unless one entire end is left open. But porch life, which can readily be carried out the year round in a suitable climate (I speak from personal experience, having slept on my porch during the coldest weather we have in this section), is the ideal life for the tuberculous subject.

It is a great mistake, and one that is frequently made, to advise a tuberculous case to go to a climate with a high relative humidity. A great many men seem to think that a mild climate is necessarily a suitable one for these cases, but such is not the fact. Tuberculous cases recover in all climates, but the recoveries in humid sections are in spite of the climate and not on account of it. Dryness is the greatest essential in a climate suitable for the treatment of phthisis, yet hundreds of cases are yearly advised to go to Florida and Southern California, both of which places (except back from the coast of California, where few go) have a relative humidity equal to that of most any place in the middle States.

It is a well-known fact that the more humid the climate the quicker a tuberculous lung breaks down, while, on the other hand, the drier the climate the slower the process, other things being equal. Dry air lessens the expectoration, improves the bronchitis, and, by so doing, diminishes the constant drain on the system, husbands the strength, and promotes sound sleep so necessary to these cases. A dry climate is necessarily one with a large per cent. of sunshine, which is the greatest of all tonics. In such a climate the extremes of heat and cold are not felt to anything like the extent they are in a moist climate, which is a great advantage in treating phthisis.

Tuberculous cases should not be advised to go to high altitudes unless they are not farther advanced than the infiltrative stage, have good hearts and fair amount of strength, and are without high temperature or nervous manifestations. Farther advanced cases than the infiltrative stage should go to medium elevations, and still farther advanced to still lower levels.

The great majority do better in a medium high altitude. Altitude is one of the greatest aids in treating phthisis; the rarefied air increases pulmonary expansion, and by so doing opens up blocked air-cells and promotes drainage. There is still some controversy as to the effect of altitude on the blood, but the evidence is in favor of there being an increase in the number of red cells, and the per cent. of hemoglobin. But as great an aid as altitude is, it must be used with discrimination, or otherwise great harm is often done.

A small, weak heart is the rule in tuberculosis, and if there is added to this much lung involvement or neurasthenia, a most common accompaniment of phthisis, the chances are that the case will not do well in a high altitude. Not nearly enough attention is given to heart-strain, which is so readily produced in the tuberculous in high altitudes, especially in those who have recently come from sea-level, and who attempt feats of endurance.

No worse advice can be given a tuberculous patient than to tell him to stay away from doctors, that medicine will do him no good. Medicine alone will not cure these cases, as every one knows, but it certainly has its place in the armamentarium of the phthisiotherapeutist, and it is foolish to decry it.

No class of cases stands in greater need of constant medical supervision than the tuberculous, and the man does not live who can treat them intelligently from a distance. All doctors in health resorts are not sharks, and a few, at least, are competent. Instead of advising these cases to stay away from doctors, they should be instructed at once to seek medical advice upon their arrival in a health resort, and to strictly follow it. If that were done in every case, the per cent. of unintentional suicides would be greatly lessened, and the per cent. of recoveries proportionately increased among the unfortunates who change climate in the search of health.

